# Reinforcement learning for individualized lung cancer screening schedules: A nested case–control study

**DOI:** 10.1002/cam4.7436

**Published:** 2024-07-01

**Authors:** Zixing Wang, Xin Sui, Wei Song, Fang Xue, Wei Han, Yaoda Hu, Jingmei Jiang

**Affiliations:** ^1^ Peking University People's Hospital Peking University Hepatology Institute, Beijing Key Laboratory of Hepatitis C and Immunotherapy for Liver Diseases Beijing China; ^2^ Department of Epidemiology and Biostatistics Institute of Basic Medical Sciences, Chinese Academy of Medical Sciences & School of Basic Medicine, Peking Union Medical College Beijing China; ^3^ Department of Radiology Peking Union Medical College Hospital Beijing China

**Keywords:** follow‐up, personalized cancer screening, pulmonary nodule, reinforcement learning

## Abstract

**Background:**

The current guidelines for managing screen‐detected pulmonary nodules offer rule‐based recommendations for immediate diagnostic work‐up or follow‐up at intervals of 3, 6, or 12 months. Customized visit plans are lacking.

**Purpose:**

To develop individualized screening schedules using reinforcement learning (RL) and evaluate the effectiveness of RL‐based policy models.

**Methods:**

Using a nested case–control design, we retrospectively identified 308 patients with cancer who had positive screening results in at least two screening rounds in the National Lung Screening Trial. We established a control group that included cancer‐free patients with nodules, matched (1:1) according to the year of cancer diagnosis. By generating 10,164 sequence decision episodes, we trained RL‐based policy models, incorporating nodule diameter alone, combined with nodule appearance (attenuation and margin) and/or patient information (age, sex, smoking status, pack‐years, and family history). We calculated rates of misdiagnosis, missed diagnosis, and delayed diagnosis, and compared the performance of RL‐based policy models with rule‐based follow‐up protocols (National Comprehensive Cancer Network guideline; China Guideline for the Screening and Early Detection of Lung Cancer).

**Results:**

We identified significant interactions between certain variables (e.g., nodule shape and patient smoking pack‐years, beyond those considered in guideline protocols) and the selection of follow‐up testing intervals, thereby impacting the quality of the decision sequence. In validation, one RL‐based policy model achieved rates of 12.3% for misdiagnosis, 9.7% for missed diagnosis, and 11.7% for delayed diagnosis. Compared with the two rule‐based protocols, the three best‐performing RL‐based policy models consistently demonstrated optimal performance for specific patient subgroups based on disease characteristics (benign or malignant), nodule phenotypes (size, shape, and attenuation), and individual attributes.

**Conclusions:**

This study highlights the potential of using an RL‐based approach that is both clinically interpretable and performance‐robust to develop personalized lung cancer screening schedules. Our findings present opportunities for enhancing the current cancer screening system.

## 1 INTRODUCTION

Tailoring treatment decisions based on patient demographics, phenotypes, and genetics, and adjusting them in response to the patient's evolving disease course is crucial for achieving precision medicine.^1^, ^2^ The reinforcement learning (RL) artificial intelligence framework is well suited for addressing this sequential decision‐making problem because it aims to train an agent to learn a policy that maximizes rewards through interactive learning.^3^ Although supervised learning algorithms dominate diagnostic testing research,^4^ reshaping diagnostic problems to fit within the RL framework can provide substantial benefits.^5^ Successful applications of RL algorithms to medical imaging tasks such as detection,^6^ segmentation,^7^ and localization^8^ have demonstrated value of this paradigm shift. However, limited research exists on RL‐based decision policies for personalized test sequences.

In the context of lung cancer screening, achieving an immediate diagnosis for small pulmonary nodules detected using low‐dose computed tomography (LDCT) is uncommon.^9^ Consequently, a considerable number of individuals undergo one or more repeat scans.^10^ The challenge lies in striking a delicate balance between the benefits of early diagnosis and the potential drawbacks of over‐investigation.^11^ Determining the appropriate criteria, duration, and frequency of follow‐up tests becomes a critical task. However, owing to inadequate evidence, the timing of these subsequent tests often falls short,^12^ resulting in substantial variations in the effectiveness of screening programs.^13^, ^14^ Similar difficulties exist when scheduling screenings for breast, cervical, and colorectal cancers.^12^

In the present study, we aimed to address the challenge of establishing an individualized screening schedule (ISS) and to investigate the potential of RL in patient visit planning. Our focus was on ensuring effective communication with physicians by emphasizing the interpretability and extrapolation aspects of the RL method. We performed a comparative analysis of the ISS devised through RL with expert‐derived rule‐based policies recommended in current guidelines, thereby providing valuable insights into the two approaches.

## 2 MATERIALS AND METHODS

### 2.1 Study population

**FIGURE 1:**
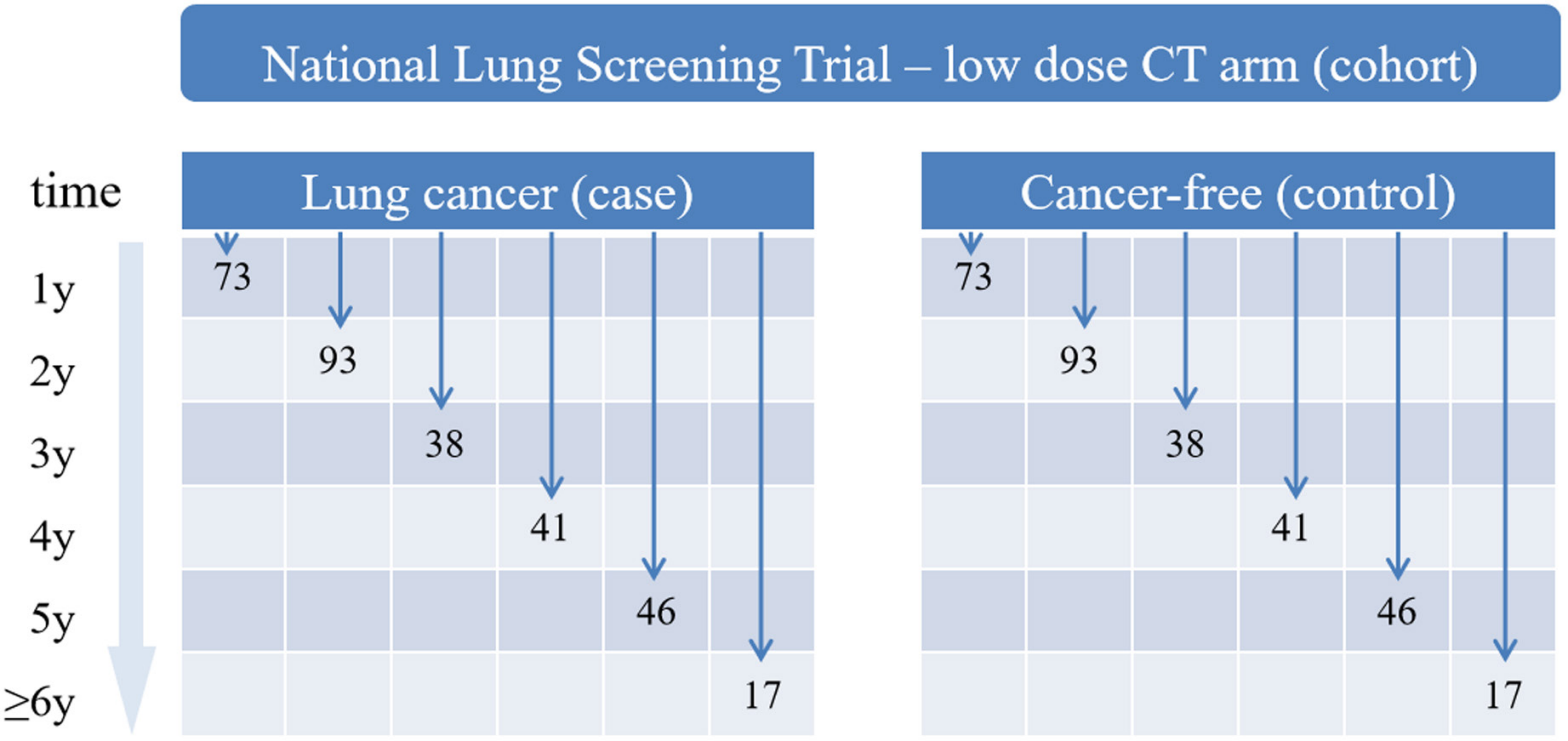
Nested case–control design. CT, computed tomography.

We obtained data from the LDCT arm of the National Lung Screening Trial (NLST),^10^ which included 26,772 individuals. Eligible participants were between the ages of 55 and 74 years and had a smoking history of over 30 pack‐years; for people who quit smoking, participants had to have quit within the past 15 years. The participants underwent annual screenings for 3 consecutive years; the maximum follow‐up duration was over 8 years. To focus on sequential testing decisions, we identified 10,417 patients who tested positive on screening in at least two rounds. A positive result was defined as the presence of at least one detected non‐calcified pulmonary nodule or mass. We established a case group comprising 308 patients with lung cancer by selecting nodules and linking their observations across different screening rounds. To create a nested case–control design, we randomly selected 308 patients from the remaining pool who were free of lung cancer and matched them to the cases according to the year of cancer diagnosis (Figure 1). This resulted in a combined sample size of 616 patients, which we further divided into a training dataset of 462 patients for training the ISS policy models and a validation dataset of 154 patients. The NLST was approved by institutional review boards of the participating centers.

### 2.2 State variables

We considered three sets of state variables for the development of the ISS.^1^ Nodule diameter, which was measured as the average of the longest diameter and the longest diameter perpendicular to it, rounded to the nearest integer. This variable is widely accepted as an important factor in existing rule‐based policies for evaluating cancer risk.^2^, ^15^, ^16^ Nodule attenuation and margin shape, which describe the appearance of the nodules and provide additional morphology information that may reflect the biological nature of the lesion. These variables were categorized as solid, part‐solid, non‐solid (ground glass opacity), and spiculated or not.^3^ Patient information, including age, sex, smoking status (former or current), smoking pack‐years, and family history of lung cancer. These are confirmed risk factors for lung cancer and may contribute to improving the individualized properties of the ISS policy models.

Notably, the nodule diameter and appearance can change over time. However, in the NLST and in practice, such information is only observed at a few time points.^17^ To account for decisions at any visit time, we used linear extrapolation for the nodule diameter and a last‐observation‐carried‐forward approach for nodule attenuation and margin interpolation.

### 2.3 Stage, action, and reward

We limited ISS decisions to a maximum of three stages for each patient. This was based on the availability of image results from the baseline and two subsequent repeat screenings, which, in most cases, provided sufficient information to make a definitive clinical judgment regarding malignancy. At each decision stage, we considered four possible choices of action: immediate diagnostic work‐up (DIAG), follow‐up LDCT in 3 months (F3), annual follow‐up LDCT (F12), or discontinuation of screening (END). These choices were in line with established rule‐based policies and were designed to simplify the decision‐making process in practical settings. A combination of these resulted in a total of 22 possible sequential scenarios (e.g., F12‐F3‐DIAG, F12‐F12‐END; not including illogical sequential scenarios, such as DIAG‐F12‐F3, F12‐END‐F12). Introducing additional decision stages or increasing the number of choices of action per stage would greatly expand the space of state–action pairs for analysis. To evaluate each sequential scenario, we used the ground truth outcome of nodules in patients, specifically the diagnosis of lung cancer and the corresponding time of diagnosis. This evaluation was conducted using a reward function defined as:

(1) $$\begin{array}{cc} R=\; & 100-70\times \text{misdiagnosis}-60\times \text{missed diagnosis}\\ & -5\times \text{delayed diagnosis}-10\times \mathrm{no}.\;\text{of tests}. \end{array}$$

where, misdiagnosis denotes DIAG for a cancer‐free patient, missed diagnosis denotes END for a patient with lung cancer, delayed diagnosis denotes a follow‐up time longer than the ground truth time for the diagnosis of lung cancer, and number of tests (range: 0–3) is used to account for the risk of repeated exposure to radiation from LDCT. We determined the weights through consultations with experts and subsequent tuning, aiming to achieve a reward range between 10 and 100.

### 2.4 Learning framework

The high temporal and spatial heterogeneity in pulmonary nodules^18^ present a challenge, with limited information available on the transition function between states. To tackle this issue, we used a temporal‐difference method called Q‐learning,^2^ which does not depend on prior knowledge. The central goal of our learning framework was to optimize a Q‐function defined as:

(2) $$Q_{t}^{*}\left(s_{t},a_{t}\right)=E\left(R_{t}+\gamma \max_{a_{t+1}}Q_{t+1}^{*}\left(s_{t+1},a_{t+1}\right)\right).$$

where, $s_{t}$ and $a_{t}$ denote observations of the state and action random variables, respectively, and γ is a discount rate. We set γ=1 such that the decision qualities at each stage are considered equally.

The optimal ISS policy given a state at any decision stage is therefore defined as:

(3) $$\pi_{t}^{*}\left(s_{t}\right)=\arg\max_{a_{t}}Q_{t}^{*}\left(s_{t},a_{t}\right).$$

The recursive form of the Q‐function in Equation (2) means that the optimal choice of an action at *t* using Equation (3) needs to be based on the best choice at *t* + 1. Therefore, $Q_{t}$ is estimated backwards from the final stage. For ease of interpretation, we used a linear model for $Q_{3}$:

(4) $$Q_{3}\left(s_{3},a_{3}\right)=E\left(R_{3}\right)=\beta_{3}+\theta_{3}S_{3}+\omega_{3}{S_{3}}^{’}A_{3}.$$

where, θ and ω denote the regression coefficient matrix for state random vector S, and interaction vectors between S and the action variable A, respectively. Notably, interaction terms are critical in Q‐learning because the objective is to identify the most effective interactions with respect to the environment (i.e., state variables) so as to optimize the final reward.

Next, we used the estimated $Q_{3}$ to model $Q_{2}$ and then $Q_{1}$ in a similar linear fashion. These equations imply that once the Q‐functions are established, the optimal action sequences are automatically determined based on the observation of the state variables. This minimal computational requirement makes this learning framework particularly advantageous in the context of cancer screening.

### 2.5 Policy models

By considering the 22 different sequential scenarios for each of the 462 patients in the training dataset, we generated a total of 10,164 episodes for training the following policy models: diameter only (Model D), diameter combined with nodule appearance (Model DN), diameter combined with patient information (Model DP), and diameter combined with both nodule appearance and patient information (Model DNP). These models were used to evaluate the value of including additional state information and to identify the most effective combination.

Nodule information is updated over stages, and therefore we considered two approaches to make use of the history of nodule diameter: using all available information directly and using the rate of change in diameter. The rate of change is defined as:

(5) $${\text{delta}}_{i}=\left({\text{diameter}}_{i}-{\text{diameter}}_{i‐1}\right)/t_{i}-t_{i‐1}.$$

For instance, in the second decision stage, we used both values of the diameter at *t*_1_ and *t*_2_ for the direct approach, and used the diameter and delta values at *t*_2_ for the delta approach. We used (Dd), (DdN), (DdP), and (DdNP) to denote the delta policy models to distinguish them from the direct approach.

### 2.6 Effect evaluation

To compare the policy models, we computed the rates of misdiagnosis, missed diagnosis, and delayed diagnosis based on their recommended action sequences. Subsequently, we identified the three policy models with the lowest rates (since lower rates are preferable) for further investigation.

We conducted a head‐to‐head comparison between the three best‐performing RL‐based policies and two expert‐based policies: the National Comprehensive Cancer Network (NCCN) Lung Cancer Screening Guideline (Version 1.2023)^15^ and the China Guideline for the Screening and Early Detection of Lung Cancer (C‐SED; version 2021).^16^ Using the validation dataset, we determined the optimal policy for each individual patient. This optimal policy was defined as the one (or multiple policies under certain conditions) that resulted in the earliest diagnostic work‐up or the shortest follow‐up duration in patients with cancer, and the shortest follow‐up duration or the shortest time for diagnostic work‐up in cancer‐free patients. Our definition of the optimal policy took into consideration the minimization of follow‐up tests and adverse event rates. Additionally, we investigated the distribution of optimal policies across different subgroups based on patient and nodule information.

### 2.7 Statistical analysis

We summarized patient characteristics using measures including mean (standard deviation), median (interquartile range), and frequency (%), as appropriate. Statistical significance was defined as a *p* < 0.05. We generated the episodes and performed basic analyses using SAS 9.4 (SAS Institute Inc., Cary, NC, USA). We implemented the Q‐learning algorithm in R 4.1.2 with the package “DynTxRegime” version 4.11 (The R Project for Statistical Computing, Vienna, Austria). The R codes for establishing and applying the personalized schedule algorithms are available in the Appendix file (Data S2).

## 3 RESULTS

### 3.1 Patient characteristics

The study included patients with a mean (standard deviation) age of 63.0 (5.2) years, of which 58.8% were men. The patients had a median (interquartile range) number of smoking pack‐years of 53 (42–73), and 48.7% had not quit smoking before participating in the NLST. The rate of a family history of lung cancer among first‐degree relatives was 25.5%. The median (interquartile range) follow‐up duration was 2155 (991–2452) days, with a significant difference of 1018 versus 2449.5 days between cases and controls. Among patients with lung cancer, the distribution of pathological stage at diagnosis was 59.1% (stage I), 4.9% (stage II), 15.3% (stage III), and 2.3% (stage IV). Table 1 provides specific information about cases and controls in the training and validation datasets.

**TABLE 1:** Patient characteristics.

	Training		Validation	
	Case	Control	Case	Control
	*n*			
Age (year), mean (SD)	63.7 (5.2)	62.1 (5.1)	63.8 (5.2)	62.5 (5.6)
Man, *n* (%)	139 (60.2)	135 (58.4)	40 (52.0)	48 (62.3)
Woman, *n* (%)	92 (39.8)	96 (41.6)	37 (48.1)	29 (37.7)
Current smoker, *n* (%)	112 (48.5)	123 (53.3)	36 (46.8)	45 (58.4)
Former smoker, *n* (%)	119 (51.5)	108 (46.8)	41 (53.3)	32 (41.6)
Smoking pack‐year, median (IQR)	56 (45–80)	49 (40–65)	61.5 (48–82)	52 (41–66)
Lung cancer family history, *n* (%)	55 (23.8)	63 (27.3)	17 (22.1)	22 (28.6)
Follow‐up duration (days), median (IQR)	975 (746–1645)	2452 (2317–2590)	1189 (790–1779)	2437 (2303–2541)
Cancer stage, *n* (%)				
Ia/Ib	131 (56.7)	‐	51 (66.2)	‐
IIa/IIb	11 (4.8)	‐	4 (5.2)	‐
IIIa/IIIb	38 (16.5)	‐	9 (11.7)	‐
V	46 (19.9)	‐	11 (14.3)	‐
Occult carcinoma/not assessed	5 (2.2)	‐	2 (2.6)	‐

Abbreviations: IQR, interquartile range; SD, standard deviation.

### 3.2 Role of state variables

**FIGURE 2:**
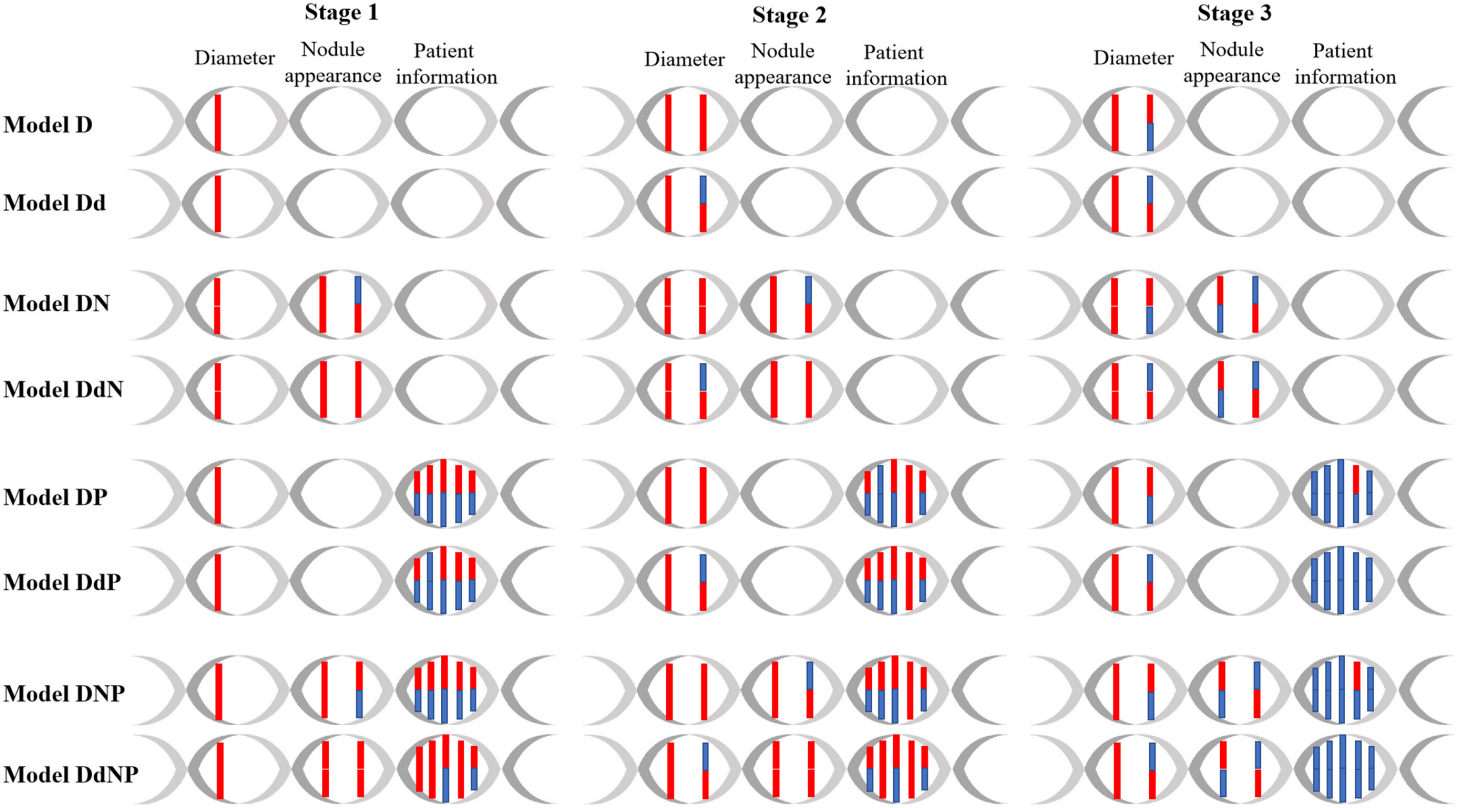
Policy models and identified roles of the state variables. Diameter variables are current and history values; nodule appearance variables are attenuation and margin shape; and patient information variables are age, sex, smoking status, smoking pack‐years, and family history of lung cancer among first‐degree relatives (arranged from left to right). Main (above) and interaction (below) effects are shown as red connections if they are statistically significant (*p* < 0.05), and blue connections otherwise; they are not shown if they are not considered in a policy model. d, delta policy models; D, diameter only; DN, diameter combined with nodule appearance; DNP, diameter combined with both nodule appearance and patient information; DP, diameter combined with patient information.

In a policy model, we can identify two roles of the state variables: main effects and interaction effects. Main effects represent independent determinants of decision quality and interaction effects act as moderators. By analyzing the statistical significance of each state variable in the trained policy models, we can illustrate these roles, as shown in Figure 2.

Irrespective of the decision stage, the current value of the nodule diameter exerted both main and interaction effects in all the policy models. However, the role of the history diameter was influenced by how this information was incorporated into the stage 3 decision. Specifically, models D, DN, DP, and DNP showed a main effect for the history diameter whereas models Dd, DdN, DdP, and DdNP demonstrated an interaction effect. However, the interaction effect of the history diameter predominated in the stage 2 decision for all policy models.

Concerning nodule appearance, attenuation demonstrated main and interaction effects in stages 1 and 2 for all policy models, but only a main effect in stage 3. Although the identification of the main effect for nodule shape was influenced by the choice of policy model used, an interaction effect was present in nearly all policy models.

All patient information variables (age, sex, smoking status, smoking pack‐years, and family history) demonstrated a main effect in stages 1 and 2 for all policy models. However, only a few of these variables exhibited an interaction effect. Remarkably, smoking pack‐years was exceptional, showing both main and interaction effects in stages 1 and 2. Moreover, smoking pack‐years was the only variable identified with a main effect in stage 3.

### 3.3 Effectiveness of the policy models

**FIGURE 3:**
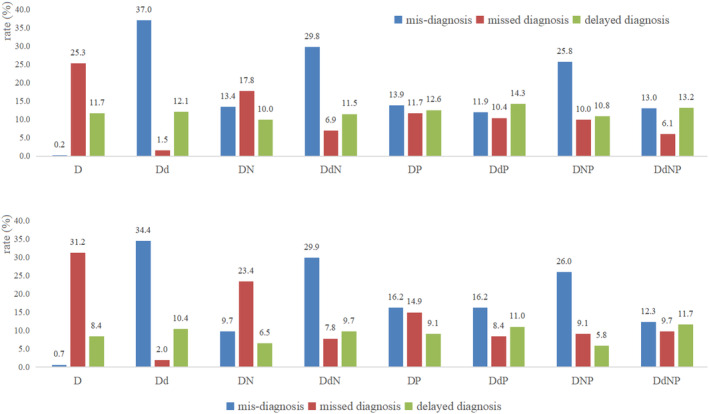
Policy model performance for the training (above) and validation (below) datasets for misdiagnosis, missed diagnosis, and delayed diagnosis.

Analysis of the rates of misdiagnosis and missed diagnosis for the policy models revealed interesting patterns (Figure 3). Model D achieved an impressively low misdiagnosis rate (training: 0.2%; validation: 0.7%), yet it had a relatively high missed diagnosis rate (training: 25.3%; validation: 31.2%). In contrast, model Dd demonstrated a higher misdiagnosis rate (training: 37.0%; validation: 34.4%) but a lower missed diagnosis rate (training: 1.5%; validation: 2.0%). These issues were significantly mitigated when other state variables were incorporated, particularly patient‐specific information. For instance, although there was a slight increase in the missed diagnosis rate compared with model Dd (from 2.0% to 7.8%, 8.4%, and 9.7% for models DdN, DdP, and DdNP, respectively), there was a substantial reduction in the misdiagnosis rate (from 34.4% to 29.9%, 16.2%, and 12.3% for models DdN, DdP, and DdNP, respectively) in validation. The policy models exhibited no significant difference in the delayed diagnosis rate.

Taking all unfavorable events into consideration, DdNP was considered the best performing model because it controlled the rates to approximately 10% (12.3%, 9.7%, and 11.7% for misdiagnosis, missed diagnosis, and delayed diagnosis, respectively) in validation. Two other models also achieved overall good performance in validation: the DdP model yielded rates of 16.2%, 8.4%, and 11.0%; the DP model yielded rates of 16.2%, 14.9%, and 9.1%.

### 3.4 Benchmark with rule‐based policies

**FIGURE 4:**
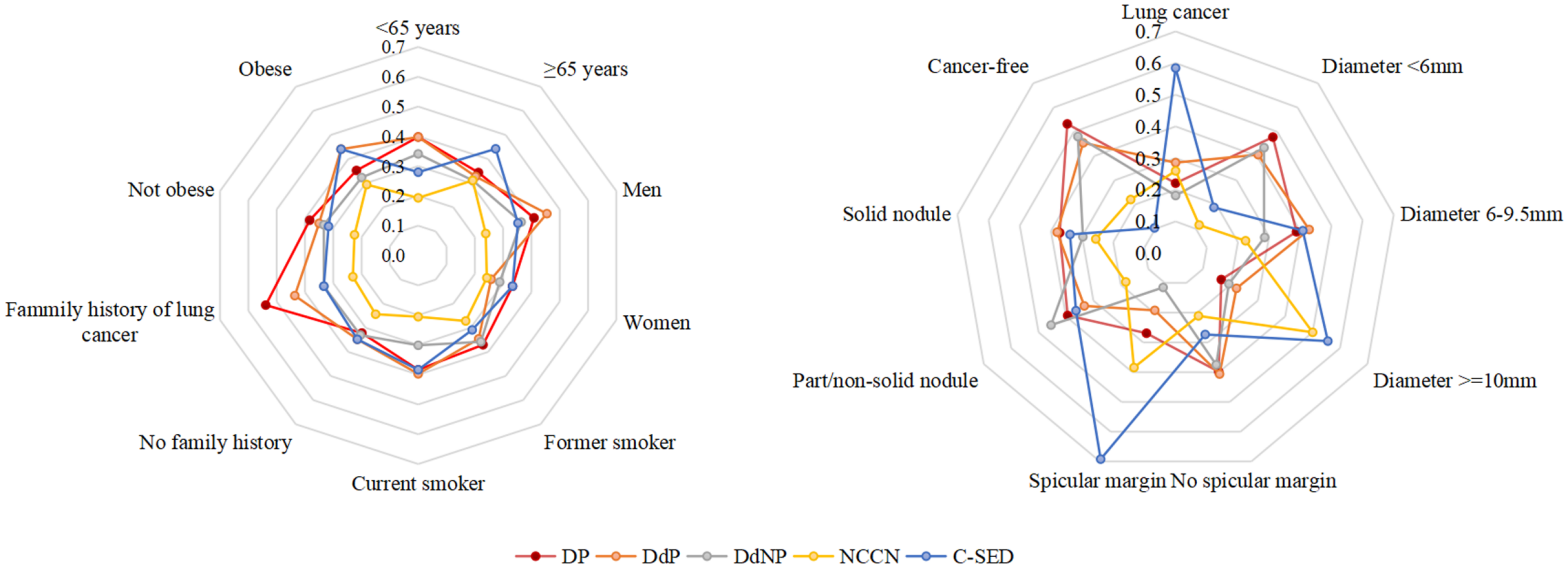
Optimal strategy identified for the patient (left) and nodule (right) subgroups. Data shown as probability of being the optimal strategy among three reinforcement learning‐based policies (models DP, DdP, DdNP) and two rule‐based policies (NCCN, C‐SED) recommended in the current guidelines. C‐SED, China Guideline for the Screening and Early Detection of Lung Cancer; d, delta policy models; DNP, diameter combined with both nodule appearance and patient information; DP, diameter combined with patient information; NCCN, National Comprehensive Cancer Network.

The head‐to‐head comparison of five policies revealed that each of the three best‐performing models (DP, DdP, and DdNP) were determined to be the optimal choice for 37.7%, 37.0%, and 33.1% of patients in the validation dataset, respectively, with probabilities being 24.0% and 34.4% for the two rule‐based policies (NCCN and C‐SED), respectively. In subgroup analyses (Figure 4), model DP outperformed other policies for patients with a family history of lung cancer, with an optimal policy selection rate of 53.9% in this group. Other demographic factors did not significantly affect the probability of selecting the optimal policy. Notably, although the C‐SED demonstrated better performance for patients with lung cancer (optimal policy for 58.4% of patients in this group), it had the disadvantage of being the least optimal policy for cancer‐free patients (10.4% compared with 53.3% [model DP], 45.5% [model DdP], 48.1% [model DdNP], and 22.1% [NCCN] of patients in this group). In contrast, all RL‐based policies exhibited robust performance across nodule characteristics, including malignancy, size, and appearance.

## 4 DISCUSSION

The rapid expansion of the population undergoing cancer screening highlights the need for effective follow‐up tests.^19^ To the best of our knowledge, this was the first study to use Q‐learning to customize patient visit plans in cancer screening. We demonstrated the clinical interpretability and performance optimality of this novel approach. There are several noteworthy findings: First, our results highlight the potential to expand the current guidelines on nodule management, which primarily focus on nodule diameter and attenuation, by incorporating variables such as nodule shape and smoking pack‐years as valuable considerations that affect decision quality both directly and by interacting with the selection of follow‐up testing intervals. Second, adverse outcomes for both patients with and without cancer can be greatly minimized by incorporating patient‐specific variables into RL‐based policy. Lastly, our RL models demonstrated validity and robustness across diverse patient subgroups based on demographics and phenotypes, presenting a practical strategy for establishing ISS and advancing personalized cancer screening.

In the realm of test sequence planning, various approaches have been proposed in research fields other than cancer screening. For instance, Bansal et al. put forward a value‐of‐information framework that considers time‐related uncertainty when determining the optimal time to collect biomarker data for patients with cystic fibrosis.^20^ Similarly, Tomer et al. adopted a joint modeling approach to predict the cumulative risk of progression in prostate cancer and used a risk threshold (5%) to decide whether surveillance tests should be conducted at future time points.^21^ However, cancer screening typically involves a more diverse population with varying underlying illnesses; as a result, designing an ISS is more challenging because this requires fewer test sequences but places a greater emphasis on accurately balancing the benefits and harms. The Q‐learning algorithm used in our study is characterized by its conceptual simplicity and computational efficiency, offering clear advantages for clinical interpretation and implementation, particularly in the setting of cancer screening. Notably, all state variables examined in this study are commonly used in clinical practice. By extending similar methodologies to encompass a broader array of data sources (including the integration of radiomics and biomarkers), there is potential to uncover avenues for enhancing the existing cancer screening system.

The outcomes of our study support our hypothesis that RL‐based policies have the capacity to outperform existing rule‐based policies. An intriguing discovery is that all of the trained policy models consistently achieved the same level of performance in the validation dataset as in the training dataset, despite the training dataset (*n* = 462) being relatively small. This can be attributed to the data augmentation process, which involved evaluating all 22 possible sequential scenarios for each patient and generating 10,164 scenario episodes. This enabled comprehensive rule mining beyond human cognitive limitations. These results demonstrate the replicability of our method and provide a solid foundation for its implementation in clinical settings.

Although our study generated important insights, several limitations deserve attention. First, the linear assumptions used when constructing the Q‐function were intended for ease of communication with clinicians but may not be as robust or effective as more advanced methods like deep RL. Second, given the limited sample size, caution must be applied when interpreting the results of the subgroup analyses. Although our analysis was based on a nationwide study, the availability for data of patients with cancer who had a sufficient number of repeat screening images to devise sequential decision rules was still limited. Lastly, the generalizability of our findings to populations beyond those eligible for the NLST (e.g., individuals aged 55–74 years with a history of heavy smoking) remains uncertain, highlighting the need for real‐world evaluations of the effectiveness of the policy models.

In summary, this study illustrates how an RL‐based approach, which is both clinically interpretable and performance‐robust, can be used to develop individually tailored lung cancer screening schedules. We also highlight the value of incorporating additional nodule and patient data that are currently not accounted for in the guidelines. These results emphasize the importance and feasibility of transitioning from rule‐based screening to a new personalized standard.

## AUTHOR CONTRIBUTIONS

**Zixing Wang:** Conceptualization (equal); formal analysis (equal); funding acquisition (equal); writing – original draft (equal). **Xin Sui:** Data curation (equal); investigation (equal); writing – review and editing (equal). **Wei Song:** Conceptualization (equal); data curation (equal); investigation (equal); resources (equal); writing – review and editing (equal). **Fang Xue:** Data curation (equal); methodology (equal); software (equal); writing – review and editing (equal). **Wei Han:** Data curation (equal); investigation (equal); software (equal); writing – review and editing (equal). **Yaoda Hu:** Data curation (equal); investigation (equal); writing – review and editing (equal). **Jingmei Jiang:** Conceptualization (equal); data curation (equal); methodology (equal); supervision (equal); writing – review and editing (equal).

## CONFLICT OF INTEREST STATEMENT

None.

## ETHICS STATEMENT

Not applicable for this analysis as only publicly available data was used. The original study was approved by institutional review boards of the participating centers.

## Supporting information


Data S1.

Data S2.

## Data Availability

Data supporting the study are available at: https://www.cancerimagingarchive.net/collection/nlst/. The analysis R code is available in the Appendix.
