# Supplementary material for: Reinforcement learning for individualized lung cancer screening schedules: A nested case–control study
Source: Cancer Med. 2024 Jul 1;13(13):e7436. doi: 10.1002/cam4.7436 (PMC11215689; doi:10.1002/cam4.7436)
Supplement: Supplementary file 2 — Data S2. [file CAM4-13-e7436-s001.docx]

**Reinforcement Learning for Individualized Lung Cancer Screening Schedules: A Nested Case-Control Study**

**Appendix: R code and model fitting results**

train_data=read.csv("your_path\\train_data.csv")

test_data =read.csv("your_path\\test_data.csv")

####Fit policy models

#### Third-Stage Analysis

# outcome model

moMain <- buildModelObj(model = ~StatD3+age+gender+cigsmok+pkyr+family+delta3+StatSo3+StatSp3,

solver.method = 'lm')

moCont <- buildModelObj(model = ~StatD3+age+gender+cigsmok+pkyr+family+delta3+StatSo3+StatSp3,

solver.method = 'lm')

fit3_ DdNP <- qLearn(moMain = moMain, moCont = moCont,

data =train, response = train$reward, txName = 'A3')

moMain <- buildModelObj(model = ~StatD3+age+gender+cigsmok+pkyr+family+delta3,

solver.method = 'lm')

moCont <- buildModelObj(model = ~StatD3+age+gender+cigsmok+pkyr+family+delta3,

solver.method = 'lm')

fit3_ DdP <- qLearn(moMain = moMain, moCont = moCont,

data =train, response = train$reward, txName = 'A3')

moMain <- buildModelObj(model = ~StatD3+age+gender+cigsmok+pkyr+family+StatD2+StatD1,

solver.method = 'lm')

moCont <- buildModelObj(model = ~StatD3+age+gender+cigsmok+pkyr+family+StatD2+StatD1,

solver.method = 'lm')

fit3 _ DP <- qLearn(moMain = moMain, moCont = moCont,

data = train, response = data$reward, txName = 'A3')

#### Second-Stage Analysis

# outcome model

moMain <- buildModelObj(model = ~StatD2+age+gender+cigsmok+pkyr+family+delta2+StatSo2+StatSp2,

solver.method = 'lm')

moCont <- buildModelObj(model = ~StatD2+age+gender+cigsmok+pkyr+family+delta2+StatSo2+StatSp2,

solver.method = 'lm')

fit2_DdNP <- qLearn(moMain = moMain, moCont = moCont,

data = train, response = fit3, txName = 'A2')

moMain <- buildModelObj(model = ~StatD2+age+gender+cigsmok+pkyr+family+delta2,

solver.method = 'lm')

moCont <- buildModelObj(model = ~StatD2+age+gender+cigsmok+pkyr+family+delta2,

solver.method = 'lm')

fit2_DdP <- qLearn(moMain = moMain, moCont = moCont,

data = train, response = fit3, txName = 'A2')

moMain <- buildModelObj(model = ~StatD2+age+gender+cigsmok+pkyr+family+StatD1,

solver.method = 'lm')

moCont <- buildModelObj(model = ~StatD2+age+gender+cigsmok+pkyr+family+StatD1,

solver.method = 'lm')

fit2_DP <- qLearn(moMain = moMain, moCont = moCont,

data = train, response = fit3, txName = 'A2')

#### First-stage Analysis

# outcome model

moMain <- buildModelObj(model = ~StatD1+age+gender+cigsmok+pkyr+family+StatSo1+StatSp1,

solver.method = 'lm')

moCont <- buildModelObj(model = ~StatD1+age+gender+cigsmok+pkyr+family+StatSo1+StatSp1,

solver.method = 'lm')

fit1_DdNP <- qLearn(moMain = moMain, moCont = moCont,

data = train, response = fit2_ DdNP, txName = 'A1')

moMain <- buildModelObj(model = ~StatD1+age+gender+cigsmok+pkyr+family,

solver.method = 'lm')

moCont <- buildModelObj(model = ~StatD1+age+gender+cigsmok+pkyr+family,

solver.method = 'lm')

fit1_DdP <- qLearn(moMain = moMain, moCont = moCont,

data = train, response = fit2_DdP, txName = 'A1')

moMain <- buildModelObj(model = ~StatD1+age+gender+cigsmok+pkyr+family,

solver.method = 'lm')

moCont <- buildModelObj(model = ~StatD1+age+gender+cigsmok+pkyr+family,

solver.method = 'lm')

fit1_DP <- qLearn(moMain = moMain, moCont = moCont,

data = DP, response = fit2_DP, txName = 'A1')

####Apply policy models

optTx(fit1_DdNP,test)$optimalTx

optTx(fit2_DdNP,test)$optimalTx

optTx(fit3_DdNP,test)$optimalTx

optTx(fit1_DdP,test)$optimalTx

optTx(fit2_DdP,test)$optimalTx

optTx(fit3_DdP,test)$optimalTx

optTx(fit1_DP,test)$optimalTx

optTx(fit2_DP,test)$optimalTx

optTx(fit3_DP,test)$optimalTx

fitted models:

**fit3_DdNP**

Coefficients:

Estimate Std. Error t value Pr(>|t|)

(Intercept) 9.798e+01 3.983e+00 24.598 < 2e-16 ***

StatD3 -5.955e-01 5.666e-02 -10.510 < 2e-16 ***

age -4.827e-02 6.149e-02 -0.785 0.432471

gender -4.213e-01 6.427e-01 -0.655 0.512181

cigsmok -6.058e-01 6.118e-01 -0.990 0.322157

pkyr 1.861e-02 1.208e-02 1.541 0.123376

family -9.530e-01 6.969e-01 -1.367 0.171527

delta3 1.207e+01 7.731e+01 0.156 0.875903

StatSo3 -2.067e+00 7.557e-01 -2.735 0.006259 **

StatSp3 -3.789e-01 8.372e-01 -0.453 0.650888

A3F12 -2.940e-01 6.608e+00 -0.044 0.964510

A3F3 8.736e+00 6.608e+00 1.322 0.186199

StatD3:A3F12 -1.271e+00 9.190e-02 -13.827 < 2e-16 ***

StatD3:A3F3 -7.697e-01 9.190e-02 -8.375 < 2e-16 ***

age:A3F12 -1.141e-01 1.020e-01 -1.119 0.263099

age:A3F3 -6.718e-02 1.020e-01 -0.659 0.509951

gender:A3F12 1.169e+00 1.066e+00 1.097 0.272775

gender:A3F3 8.667e-02 1.066e+00 0.081 0.935199

cigsmok:A3F12 -1.344e+00 1.015e+00 -1.325 0.185274

cigsmok:A3F3 -3.675e-01 1.015e+00 -0.362 0.717167

pkyr:A3F12 -1.780e-02 2.004e-02 -0.888 0.374318

pkyr:A3F3 -6.278e-03 2.004e-02 -0.313 0.754063

family:A3F12 -7.606e-01 1.155e+00 -0.658 0.510363

family:A3F3 -7.247e-01 1.155e+00 -0.627 0.530525

delta3:A3F12 -4.024e+02 1.096e+02 -3.672 0.000242 ***

delta3:A3F3 -4.651e+01 1.096e+02 -0.424 0.671278

StatSo3:A3F12 6.872e-01 1.259e+00 0.546 0.585064

StatSo3:A3F3 -3.992e-01 1.259e+00 -0.317 0.751122

StatSp3:A3F12 -6.561e+00 1.394e+00 -4.706 2.58e-06 ***

StatSp3:A3F3 -4.147e+00 1.394e+00 -2.974 0.002947 **

---

Signif. codes: 0 ‘***’ 0.001 ‘**’ 0.01 ‘*’ 0.05 ‘.’ 0.1 ‘ ’ 1

Residual standard error: 17.19 on 6900 degrees of freedom

Multiple R-squared: 0.317, Adjusted R-squared: 0.3141

F-statistic: 110.4 on 29 and 6900 DF, p-value: < 2.2e-16

**fit2_DdNP**

Coefficients:

Estimate Std. Error t value Pr(>|t|)

(Intercept) 1.006e+02 5.181e-01 194.127 < 2e-16 ***

StatD2 -6.744e-01 8.006e-03 -84.235 < 2e-16 ***

age -6.444e-02 8.003e-03 -8.052 9.53e-16 ***

gender -4.813e-01 8.371e-02 -5.750 9.33e-09 ***

cigsmok -7.440e-01 7.961e-02 -9.346 < 2e-16 ***

pkyr 1.633e-02 1.570e-03 10.403 < 2e-16 ***

family -1.200e+00 9.078e-02 -13.222 < 2e-16 ***

delta2 -4.612e-02 8.483e-01 -0.054 0.95665

StatSo2 -2.375e+00 9.762e-02 -24.332 < 2e-16 ***

StatSp2 -6.236e-01 1.082e-01 -5.761 8.71e-09 ***

A2F12 -1.223e+00 6.347e-01 -1.927 0.05407 .

A2F3 -1.118e+00 6.347e-01 -1.762 0.07819 .

StatD2:A2F12 2.299e-02 9.903e-03 2.322 0.02028 *

StatD2:A2F3 3.911e-02 9.903e-03 3.949 7.92e-05 ***

age:A2F12 -8.189e-03 9.804e-03 -0.835 0.40361

age:A2F3 2.828e-03 9.804e-03 0.288 0.77305

gender:A2F12 3.162e-01 1.025e-01 3.084 0.00205 **

gender:A2F3 1.209e-01 1.025e-01 1.179 0.23834

cigsmok:A2F12 3.871e-02 9.750e-02 0.397 0.69135

cigsmok:A2F3 6.522e-02 9.750e-02 0.669 0.50355

pkyr:A2F12 -5.917e-03 1.923e-03 -3.077 0.00210 **

pkyr:A2F3 -6.675e-04 1.923e-03 -0.347 0.72852

family:A2F12 6.084e-02 1.112e-01 0.547 0.58428

family:A2F3 7.491e-02 1.112e-01 0.674 0.50050

delta2:A2F12 7.735e+00 9.825e-01 7.873 4.01e-15 ***

delta2:A2F3 1.646e+00 9.825e-01 1.675 0.09400 .

StatSo2:A2F12 9.546e-01 1.201e-01 7.948 2.19e-15 ***

StatSo2:A2F3 2.934e-01 1.201e-01 2.443 0.01460 *

StatSp2:A2F12 -6.963e-01 1.327e-01 -5.249 1.58e-07 ***

StatSp2:A2F3 -1.454e-01 1.327e-01 -1.096 0.27306

---

Signif. codes: 0 ‘***’ 0.001 ‘**’ 0.01 ‘*’ 0.05 ‘.’ 0.1 ‘ ’ 1

Residual standard error: 1.463 on 6900 degrees of freedom

Multiple R-squared: 0.85, Adjusted R-squared: 0.8494

F-statistic: 1348 on 29 and 6900 DF, p-value: < 2.2e-16

**fit1_DdNP**

Coefficients:

Estimate Std. Error t value Pr(>|t|)

(Intercept) 100.539300 1.253051 80.236 < 2e-16 ***

StatD1 -0.666291 0.019377 -34.385 < 2e-16 ***

age -0.064441 0.019341 -3.332 0.000867 ***

gender -0.484671 0.202978 -2.388 0.016976 *

cigsmok -0.746519 0.192596 -3.876 0.000107 ***

pkyr 0.016329 0.003797 4.301 1.72e-05 ***

family -1.200149 0.219726 -5.462 4.87e-08 ***

StatSo1 -2.365005 0.232169 -10.187 < 2e-16 ***

StatSp1 -0.632313 0.262873 -2.405 0.016181 *

A1F12 -1.065935 1.339568 -0.796 0.426216

A1F3 -1.901383 1.339568 -1.419 0.155828

StatD1:A1F12 0.190115 0.020715 9.178 < 2e-16 ***

StatD1:A1F3 0.186966 0.020715 9.026 < 2e-16 ***

age:A1F12 -0.046739 0.020677 -2.260 0.023822 *

age:A1F3 -0.023464 0.020677 -1.135 0.256500

gender:A1F12 0.426802 0.216993 1.967 0.049236 *

gender:A1F3 0.410671 0.216993 1.893 0.058459 .

cigsmok:A1F12 0.091040 0.205893 0.442 0.658381

cigsmok:A1F3 0.085914 0.205893 0.417 0.676489

pkyr:A1F12 -0.008095 0.004059 -1.995 0.046134 *

pkyr:A1F3 -0.001148 0.004059 -0.283 0.777362

family:A1F12 0.090772 0.234897 0.386 0.699189

family:A1F3 0.211462 0.234897 0.900 0.368029

StatSo1:A1F12 1.776241 0.248199 7.157 9.13e-13 ***

StatSo1:A1F3 1.067682 0.248199 4.302 1.72e-05 ***

StatSp1:A1F12 -1.488727 0.281023 -5.298 1.21e-07 ***

StatSp1:A1F3 -0.882214 0.281023 -3.139 0.001701 **

---

Signif. codes: 0 ‘***’ 0.001 ‘**’ 0.01 ‘*’ 0.05 ‘.’ 0.1 ‘ ’ 1

Residual standard error: 2.043 on 6903 degrees of freedom

Multiple R-squared: 0.6508, Adjusted R-squared: 0.6495

F-statistic: 494.9 on 26 and 6903 DF, p-value: < 2.2e-16

**fit3_DdP**

Coefficients:

Estimate Std. Error t value Pr(>|t|)

(Intercept) 9.667e+01 3.978e+00 24.303 < 2e-16 ***

StatD3 -5.610e-01 5.297e-02 -10.591 < 2e-16 ***

age -6.279e-02 6.153e-02 -1.021 0.307498

gender -1.269e-01 6.350e-01 -0.200 0.841557

cigsmok -7.134e-01 6.124e-01 -1.165 0.244094

pkyr 1.801e-02 1.214e-02 1.484 0.137894

family -9.708e-01 7.000e-01 -1.387 0.165548

delta3 5.370e+00 7.767e+01 0.069 0.944879

A3F12 7.168e-02 6.597e+00 0.011 0.991331

A3F3 8.441e+00 6.597e+00 1.280 0.200712

StatD3:A3F12 -1.393e+00 8.510e-02 -16.372 < 2e-16 ***

StatD3:A3F3 -8.314e-01 8.510e-02 -9.769 < 2e-16 ***

age:A3F12 -1.024e-01 1.021e-01 -1.004 0.315643

age:A3F3 -6.576e-02 1.021e-01 -0.644 0.519347

gender:A3F12 8.827e-01 1.053e+00 0.838 0.401886

gender:A3F3 3.063e-02 1.053e+00 0.029 0.976790

cigsmok:A3F12 -1.746e+00 1.015e+00 -1.720 0.085524 .

cigsmok:A3F3 -6.475e-01 1.015e+00 -0.638 0.523723

pkyr:A3F12 -2.008e-02 2.014e-02 -0.997 0.318775

pkyr:A3F3 -8.191e-03 2.014e-02 -0.407 0.684200

family:A3F12 -6.466e-01 1.161e+00 -0.557 0.577606

family:A3F3 -6.655e-01 1.161e+00 -0.573 0.566532

delta3:A3F12 -4.204e+02 1.101e+02 -3.819 0.000135 ***

delta3:A3F3 -5.972e+01 1.101e+02 -0.543 0.587480

---

Signif. codes: 0 ‘***’ 0.001 ‘**’ 0.01 ‘*’ 0.05 ‘.’ 0.1 ‘ ’ 1

Residual standard error: 17.28 on 6906 degrees of freedom

Multiple R-squared: 0.309, Adjusted R-squared: 0.3067

F-statistic: 134.3 on 23 and 6906 DF, p-value: < 2.2e-16

**fit2_DdP**

Coefficients:

Estimate Std. Error t value Pr(>|t|)

(Intercept) 98.5296402 0.4833708 203.839 < 2e-16 ***

StatD2 -0.6365345 0.0071254 -89.333 < 2e-16 ***

age -0.0726324 0.0074736 -9.719 < 2e-16 ***

gender -0.1888282 0.0772626 -2.444 0.0146 *

cigsmok -0.8908666 0.0744161 -11.971 < 2e-16 ***

pkyr 0.0160396 0.0014740 10.882 < 2e-16 ***

family -1.1836304 0.0850598 -13.915 < 2e-16 ***

delta2 -0.1154361 0.7964819 -0.145 0.8848

A2F12 -0.7198594 0.5921607 -1.216 0.2242

A2F3 -1.0836866 0.5921607 -1.830 0.0673 .

StatD2:A2F12 0.0029855 0.0087549 0.341 0.7331

StatD2:A2F3 0.0424458 0.0087549 4.848 1.27e-06 ***

age:A2F12 -0.0013491 0.0091546 -0.147 0.8828

age:A2F3 0.0043667 0.0091546 0.477 0.6334

gender:A2F12 0.1368466 0.0946435 1.446 0.1482

gender:A2F3 0.0937819 0.0946435 0.991 0.3218

cigsmok:A2F12 0.0057973 0.0911403 0.064 0.9493

cigsmok:A2F3 0.0840662 0.0911403 0.922 0.3564

pkyr:A2F12 -0.0045558 0.0018054 -2.523 0.0116 *

pkyr:A2F3 -0.0003311 0.0018054 -0.183 0.8545

family:A2F12 0.1366709 0.1042123 1.311 0.1897

family:A2F3 0.1174781 0.1042123 1.127 0.2597

delta2:A2F12 7.2601374 0.9223811 7.871 4.05e-15 ***

delta2:A2F3 1.5625575 0.9223811 1.694 0.0903 .

---

Signif. codes: 0 ‘***’ 0.001 ‘**’ 0.01 ‘*’ 0.05 ‘.’ 0.1 ‘ ’ 1

Residual standard error: 1.374 on 6906 degrees of freedom

Multiple R-squared: 0.8549, Adjusted R-squared: 0.8544

F-statistic: 1769 on 23 and 6906 DF, p-value: < 2.2e-16

**fit1_DdP**

Coefficients:

Estimate Std. Error t value Pr(>|t|)

(Intercept) 98.495699 1.215224 81.051 < 2e-16 ***

StatD1 -0.626287 0.018445 -33.954 < 2e-16 ***

age -0.072782 0.018792 -3.873 0.000108 ***

gender -0.194644 0.194549 -1.000 0.317109

cigsmok -0.890629 0.187245 -4.756 2.01e-06 ***

pkyr 0.016094 0.003708 4.340 1.45e-05 ***

family -1.181162 0.213901 -5.522 3.47e-08 ***

A1F12 -0.862803 1.299129 -0.664 0.506623

A1F3 -1.418323 1.299129 -1.092 0.274981

StatD1:A1F12 0.140322 0.019719 7.116 1.22e-12 ***

StatD1:A1F3 0.158541 0.019719 8.040 1.05e-15 ***

age:A1F12 -0.022266 0.020089 -1.108 0.267751

age:A1F3 -0.012699 0.020089 -0.632 0.527342

gender:A1F12 0.181191 0.207981 0.871 0.383683

gender:A1F3 0.180995 0.207981 0.870 0.384195

cigsmok:A1F12 0.047110 0.200174 0.235 0.813946

cigsmok:A1F3 0.091396 0.200174 0.457 0.647985

pkyr:A1F12 -0.006891 0.003964 -1.738 0.082202 .

pkyr:A1F3 -0.001010 0.003964 -0.255 0.798872

family:A1F12 0.294806 0.228670 1.289 0.197364

family:A1F3 0.272549 0.228670 1.192 0.233345

---

Signif. codes: 0 ‘***’ 0.001 ‘**’ 0.01 ‘*’ 0.05 ‘.’ 0.1 ‘ ’ 1

Residual standard error: 1.995 on 6909 degrees of freedom

Multiple R-squared: 0.6363, Adjusted R-squared: 0.6353

F-statistic: 604.4 on 20 and 6909 DF, p-value: < 2.2e-16

**fit3_DP**

Coefficients:

Estimate Std. Error t value Pr(>|t|)

(Intercept) 96.249815 3.925878 24.517 < 2e-16 ***

StatD3 -1.825892 0.154035 -11.854 < 2e-16 ***

age -0.079147 0.060711 -1.304 0.192388

gender -0.396450 0.628456 -0.631 0.528171

cigsmok -0.488180 0.604541 -0.808 0.419394

pkyr 0.023832 0.011991 1.987 0.046913 *

family -1.028248 0.691432 -1.487 0.137027

StatD2 1.249167 0.185482 6.735 1.77e-11 ***

StatD1 0.317009 0.092137 3.441 0.000584 ***

A3F12 -0.030311 6.511431 -0.005 0.996286

A3F3 7.788020 6.511431 1.196 0.231717

StatD3:A3F12 -1.220441 0.219098 -5.570 2.64e-08 ***

StatD3:A3F3 -0.750349 0.219098 -3.425 0.000619 ***

age:A3F12 -0.115745 0.100692 -1.149 0.250393

age:A3F3 -0.070968 0.100692 -0.705 0.480955

gender:A3F12 1.019942 1.042285 0.979 0.327830

gender:A3F3 0.091118 1.042285 0.087 0.930339

cigsmok:A3F12 -1.811784 1.002555 -1.807 0.070780 .

cigsmok:A3F3 -0.602616 1.002555 -0.601 0.547806

pkyr:A3F12 -0.017548 0.019897 -0.882 0.377847

pkyr:A3F3 -0.006281 0.019897 -0.316 0.752277

family:A3F12 -0.349197 1.146874 -0.304 0.760773

family:A3F3 -0.508281 1.146874 -0.443 0.657643

StatD2:A3F12 0.029788 0.274524 0.109 0.913596

StatD2:A3F3 0.159082 0.274524 0.579 0.562281

StatD1:A3F12 -0.189444 0.148036 -1.280 0.200688

StatD1:A3F3 -0.165789 0.148036 -1.120 0.262786

---

Signif. codes: 0 ‘***’ 0.001 ‘**’ 0.01 ‘*’ 0.05 ‘.’ 0.1 ‘ ’ 1

Residual standard error: 17.04 on 6903 degrees of freedom

Multiple R-squared: 0.328, Adjusted R-squared: 0.3254

F-statistic: 129.6 on 26 and 6903 DF, p-value: < 2.2e-16

**fit2_DP**

Coefficients:

Estimate Std. Error t value Pr(>|t|)

(Intercept) 97.739937 1.467416 66.607 < 2e-16 ***

StatD2 -0.625324 0.036097 -17.324 < 2e-16 ***

age -0.088224 0.022694 -3.888 0.000102 ***

gender -0.442109 0.234937 -1.882 0.059903 .

cigsmok -0.622674 0.226024 -2.755 0.005886 **

pkyr 0.022466 0.004478 5.017 5.38e-07 ***

family -1.175326 0.258417 -4.548 5.50e-06 ***

StatD1 0.306245 0.037568 8.152 4.23e-16 ***

A2F12 1.494182 1.797394 0.831 0.405830

A2F3 -0.520835 1.797394 -0.290 0.771999

StatD2:A2F12 -0.182920 0.042461 -4.308 1.67e-05 ***

StatD2:A2F3 -0.028286 0.042461 -0.666 0.505335

age:A2F12 -0.020245 0.027798 -0.728 0.466451

age:A2F3 0.002019 0.027798 0.073 0.942104

gender:A2F12 0.014009 0.287772 0.049 0.961176

gender:A2F3 0.106893 0.287772 0.371 0.710314

cigsmok:A2F12 -0.180519 0.276826 -0.652 0.514357

cigsmok:A2F3 0.071894 0.276826 0.260 0.795097

pkyr:A2F12 -0.017693 0.005485 -3.226 0.001263 **

pkyr:A2F3 -0.003432 0.005485 -0.626 0.531507

family:A2F12 -0.134036 0.316564 -0.423 0.672010

family:A2F3 -0.004472 0.316564 -0.014 0.988728

StatD1:A2F12 0.112154 0.044115 2.542 0.011033 *

StatD1:A2F3 0.039095 0.044115 0.886 0.375535

---

Signif. codes: 0 ‘***’ 0.001 ‘**’ 0.01 ‘*’ 0.05 ‘.’ 0.1 ‘ ’ 1

Residual standard error: 4.172 on 6906 degrees of freedom

Multiple R-squared: 0.3277, Adjusted R-squared: 0.3254

F-statistic: 146.3 on 23 and 6906 DF, p-value: < 2.2e-16

**fit1_DP**

Coefficients:

Estimate Std. Error t value Pr(>|t|)

(Intercept) 97.7399371 1.2963845 75.394 < 2e-16 ***

StatD1 -0.3190785 0.0196773 -16.216 < 2e-16 ***

age -0.0882237 0.0200470 -4.401 1.09e-05 ***

gender -0.4421093 0.2075420 -2.130 0.03319 *

cigsmok -0.6226743 0.1997507 -3.117 0.00183 **

pkyr 0.0224660 0.0039560 5.679 1.41e-08 ***

family -1.1753260 0.2281865 -5.151 2.67e-07 ***

A1F12 -0.8150998 1.3858934 -0.588 0.55646

A1F3 -1.4014646 1.3858934 -1.011 0.31194

StatD1:A1F12 0.1343665 0.0210359 6.387 1.80e-10 ***

StatD1:A1F3 0.1690807 0.0210359 8.038 1.07e-15 ***

age:A1F12 -0.0221974 0.0214312 -1.036 0.30035

age:A1F3 -0.0160023 0.0214312 -0.747 0.45528

gender:A1F12 0.1821351 0.2218717 0.821 0.41173

gender:A1F3 0.1976568 0.2218717 0.891 0.37304

cigsmok:A1F12 0.0370911 0.2135425 0.174 0.86211

cigsmok:A1F3 0.1001738 0.2135425 0.469 0.63901

pkyr:A1F12 -0.0069226 0.0042291 -1.637 0.10170

pkyr:A1F3 -0.0002219 0.0042291 -0.052 0.95815

family:A1F12 0.2881391 0.2439416 1.181 0.23757

family:A1F3 0.2848437 0.2439416 1.168 0.24298

---

Signif. codes: 0 ‘***’ 0.001 ‘**’ 0.01 ‘*’ 0.05 ‘.’ 0.1 ‘ ’ 1

Residual standard error: 2.129 on 6909 degrees of freedom

Multiple R-squared: 0.2779, Adjusted R-squared: 0.2758

F-statistic: 132.9 on 20 and 6909 DF, p-value: < 2.2e-16
